# Spatial justice and urban metabolism in arid cities: a comparative analysis with Amsterdam

**DOI:** 10.1038/s41598-026-56555-w

**Published:** 2026-06-04

**Authors:** Anas Tuffaha, Chenyu Du, Ágnes Sallay

**Affiliations:** Hungarian University of Agriculture and Life Sciences, Villányi út 29-43, Budapest, 1118 Hungary

**Keywords:** Urban metabolism, Spatial justice, Arid cities, Green infrastructure, Comparative urban analysis, Environmental social sciences, Environmental studies, Geography, Geography

## Abstract

Urban metabolism (UM) frameworks have traditionally prioritized technical efficiency metrics, often overlooking the socio-spatial distribution of metabolic benefits and burdens [1]. This gap is particularly significant in arid cities, where resource constraints amplify the consequences of inequitable green infrastructure (GI) distribution. This study examines the relationship between spatial justice (specifically regarding GI distribution) and urban metabolic patterns, centering on arid cities that face distinct metabolic challenges. The theoretical framework positions green infrastructure as a socio-ecological mediator within the urban metabolic system, connecting water, energy, and heat flows to lived environmental outcomes across socio-economic gradients. The study focuses on Amman and Cairo as representative arid cities varied in metabolic performance, by putting under comparative analysis with Amsterdam, a city recognized for integrated metabolic governance [2], we investigate how spatial equity patterns relate to broader urban metabolic characteristics by using a geospatial methodology integrating Normalized Difference Vegetation Index (NDVI) data with income-density stratification employed to analyze vegetation distribution across socio-economic gradients in all three cities. Our findings reveal pronounced disparities in relation to metabolic performance, with Disparity Ratios of 1.56 (Amman) and 2.68 (Cairo) compared to 1.10 in Amsterdam. These distribution patterns are examined in relation to each city’s documented metabolic context, governance approaches, and climatic constraints. The research positions us to understand how spatial inequality intersects with metabolic stress and demonstrates how the Disparity Ratio can function as an early-warning diagnostic of metabolic imbalance. By using a successful metabolic city, Amsterdam as an external reference point, the study identifies potential relationships between spatial equity and metabolic coherence that may inform more equitable planning approaches in resource-constrained urban contexts.

**Supplementary Information:**

The online version contains supplementary material available at 10.1038/s41598-026-56555-w.

## Introduction

Cities function as dynamic socio-ecological systems that continuously transform energy, materials, water, and information through complex metabolic processes. Since Wolman’s foundational conceptualization of urban metabolism^1^, the field has developed sophisticated methodologies for quantifying material flows and assessing urban ecological footprints^2,3^. These approaches have yielded valuable insights into urban resource efficiency, carbon emissions, and waste generation patterns across diverse global contexts.

A persistent limitation in urban metabolism literature is the reliance on aggregate, city-wide metrics that obscure significant variation in how different urban neighborhoods and populations experience metabolic processes. As noted in recent critical assessments^4^, traditional metabolic accounting tends to privilege technical efficiency over distributive justice, overlooking how environmental benefits and burdens are unevenly distributed across socio-economic gradients.

This study addresses this gap by examining the relationship between spatial justice and urban metabolic patterns, focusing on green infrastructure as an element that both influences and reflects multiple metabolic flows. Green infrastructure (encompassing vegetation, parks, street trees, and other natural elements) plays crucial roles in urban microclimate regulation, hydrological processes, air quality, and human well-being meaning its deeply connected with flows that are usually studied by Urban metabolism^2^. Its distribution across urban landscapes rarely is uniform, often reflecting historical development patterns, governance priorities, land valuation, and socio-economic stratification.

The research is situated within emerging issues that seek to bridge urban metabolism with urban political ecology and spatial justice frameworks^5,6^. Rather than treating spatial equity as merely a social outcome separate from metabolic efficiency, we explore how distributional patterns of environmental resources relate to broader urban metabolic characteristics. This integrative perspective is especially relevant for rapidly urbanizing regions and resource-constrained cities, where equitable access to ecological services is both a social imperative and a key determinant of resilience.

Our investigation uses a comparative case study approach, examining three cities representing distinct metabolic contexts and governance regimes: Amsterdam (integrated planning and circular economy ambitions), Cairo (a high-density arid megacity with documented metabolic stresses), and Amman (a rapidly growing arid city with understudied metabolic patterns). This selection enables analysis of how the relationship between GI distribution and metabolic characteristics manifests across different urban conditions, particularly in GI-deficient countries within the MENA region.

The study makes several interconnected contributions: it advances urban metabolism theory by incorporating spatial equity considerations into UM frameworks; provides a replicable geospatial methodology for analyzing socio-spatial dimensions of metabolic flows; offers empirical insights from Amman as an underrepresented case of arid city metabolism; and demonstrates how spatial diagnostics can complement traditional material flow analysis.

## Literature review

### Urban metabolism: from biophysical accounting to spatial integration

The conceptual foundations of urban metabolism trace to the mid-twentieth century, when Wolman first quantified the material inputs and outputs of a hypothetical city, establishing a framework for analyzing cities as metabolic systems^1^. This biophysical accounting tradition was advanced through Material Flow Analysis (MFA) and Substance Flow Analysis (SFA), enabling more precise tracking of material stocks and flows through urban systems^3,7^. Subsequent methodological innovations expanded UM’s scope: Life Cycle Assessment introduced temporal and systemic perspectives, while GIS and remote sensing enabled spatialization of metabolic data^8^. The shift of UM studies from measuring resources or flows to broader frameworks like social systems, economics and circular metabolism is clearly noted in Tillies publications^9^.

A significant milestone was Kennedy’s development of comparative indicators for global megacities^2^, enabling systematic comparison of metabolic performance across diverse urban contexts. Despite these advances, a systematic review of influential UM publications reveals a persistent emphasis on biophysical accounting, with fewer than 25% of highly cited studies meaningfully engaging with socio-economic or equity dimensions — a gap this research seeks to address^4^.

More recent contributions have begun to bridge this divide. Cerreta et al.^10^ demonstrates how urban metabolism frameworks can be integrated with multi-criteria spatial evaluation to support planning decisions, explicitly linking metabolic performance to urban equity outcomes. Similarly, work on peri-urban and transitional contexts has highlighted the importance of local governance and informality in shaping metabolic flows^11^. These contributions reinforce the need to move beyond aggregate efficiency indicators toward spatially sensitive and distributive approaches to urban metabolic analysis.

### Spatial justice and the urban environment

The concept of spatial justice evolved through several decades of critical urban scholarship, tracing its philosophical roots to Lefebvre’s “right to the city”^12^ and finding contemporary expression in the work of Soja^13^, Harvey^12,13^, and others. Spatial justice frameworks emphasize that the organization of urban space both reflects and reproduces social power relations, with significant implications for access to resources, services, and environmental quality.

Applied to urban environments, spatial justice concerns how environmental benefits (clean air, cooling shade, recreational space) and burdens (pollution, heat exposure, flood risk) are distributed across different neighborhoods and populations. Empirical research across multiple continents has documented consistent patterns of environmental inequality, with marginalized communities frequently experiencing disproportionate exposure to hazards and reduced access to amenities^14–16^.

Urban political ecology scholarship has been particularly influential in connecting these distributive patterns to broader political-economic processes. As Swyngedouw^6^ and Heynen^17^ argued, urban environments are produced through socio-ecological processes that are inherently political, shaped by governance structures, market forces, and historical legacies of development. From this perspective, uneven vegetation distribution represents not merely an ecological pattern but a material expression of underlying socio-political dynamics.

### Green infrastructure as metabolic mediator and justice indicator

Green infrastructure occupies a unique analytical position at the intersection of metabolic flows and spatial justice considerations^18^. Functionally, GI serves as a “crossflow element,” simultaneously influencing multiple metabolic domains: microclimate through evapotranspiration and shading^19,20^; hydrology through interception, infiltration, and stormwater management^21^; air quality through particulate filtration^22^; and human well-being through recreational and psychological benefits^23^.

GI distribution patterns often reflect and reinforce socio-spatial inequalities this is not new and could be traced to many different studies. In US cities, historical practices created enduring tree-canopy gaps along racial and income lines^24^. In European cities, despite generally higher overall greening, concerns about “green gentrification” highlight how environmental improvements can exacerbate displacement pressures^25^. In Global South megacities, fragmentation between formal and informal urban development produces stark vegetation disparities^26^.

This dual characteristic — as both metabolic mediator and justice indicator — makes GI particularly valuable for examining the relationship between spatial equity and urban metabolic patterns. However, existing research often treats these dimensions separately: UM studies quantify GI’s aggregate contribution to ecosystem services without analyzing distribution, while environmental justice studies document vegetation disparities without connecting them to broader metabolic contexts. The present study seeks to bridge this divide.

### Urban metabolism in arid cities and the global South

A significant portion of UM literature has focused on cities in temperate climates and high-income countries, with limited attention to arid regions. This represents an important knowledge gap, as cities in water-scarce regions face distinct metabolic challenges. Existing research on arid city metabolism has tended toward sectoral approaches, examining water^27^, energy for cooling^28^, or urban heat islands^29^ in isolation, with few integrated metabolic or distributional analyses.

The Middle East and North Africa region, despite containing some of the world’s most water-stressed cities, remains notably underrepresented in comparative UM literature. This research addresses this geographical gap by centering Amman as a primary case while maintaining comparative perspective with Cairo and Amsterdam.

With that both Amman and Cairo’s metabolic performance is noted to be under the expected level in comparison with Amsterdam, as noted in previous studies and analysis like Kennedys analysis to Cairo^30^, or a short analysis regarding Amman’s overall metabolic performance^31^. The focus here with the NDVI-based methodology adopted here is particularly suited to arid contexts, where the scarcity of vegetation amplifies the distributional significance of even small differences in green cover.d.

## Methodology

### Conceptual framework

**Fig. 1:**
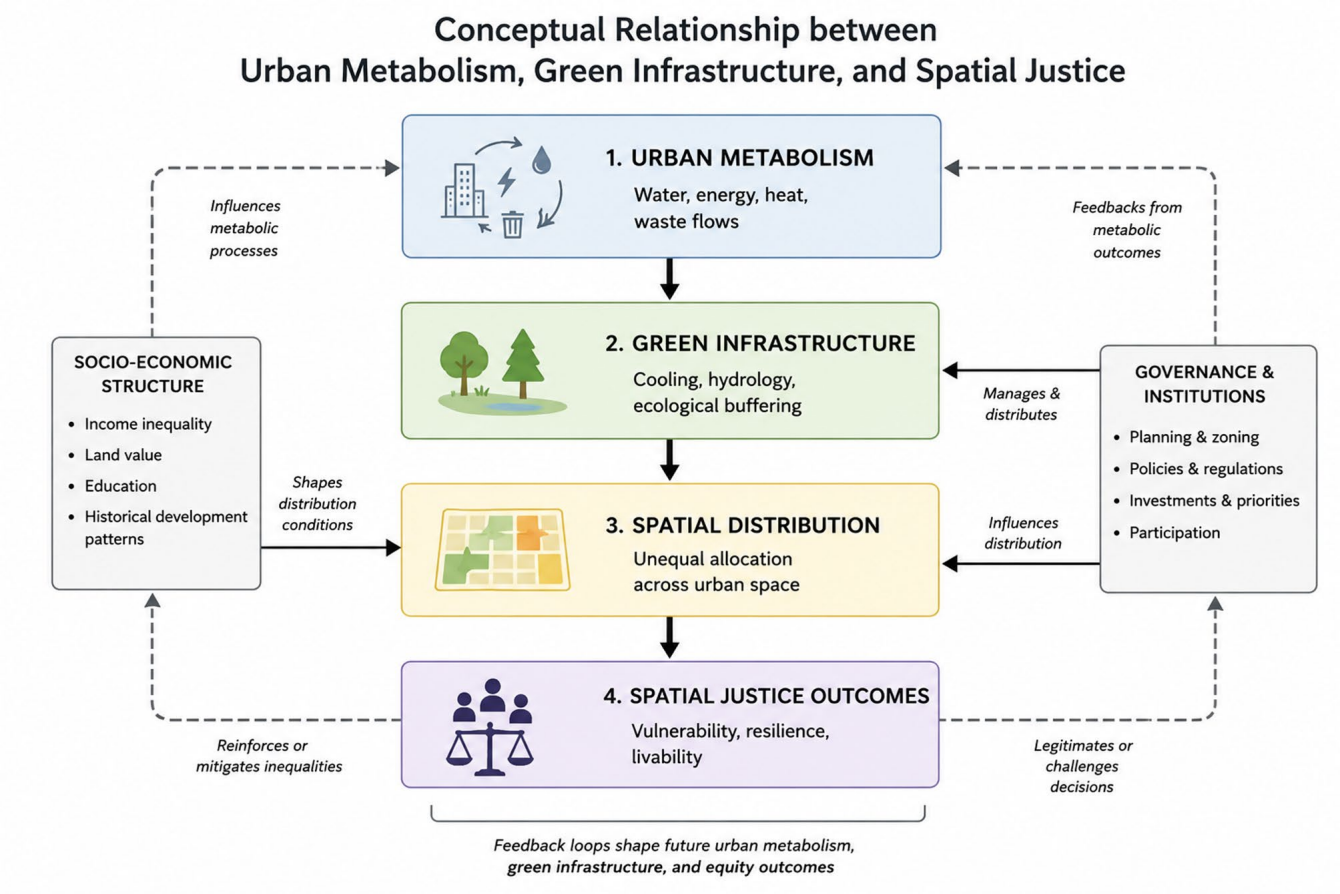
Conceptual relationship between urban metabolism, green infrastructure, and spatial justice. Solid arrows represent direct flows; dashed arrows indicate feedback loops. Governance and socio-economic structure shape both the distribution of GI and the justice outcomes experienced across urban populations.

Before presenting the analytical approach, it is important to clarify the conceptual relationship between urban metabolism, green infrastructure, and spatial justice that underpins this study. Figure 1 illustrates this framework. Urban metabolism — encompassing flows of water, energy, heat, and waste — directly shapes the need for and performance of green infrastructure. GI acts as a socio-ecological mediator, contributing to cooling, hydrological management, and ecological buffering. However, these metabolic benefits are not evenly distributed across urban space. The spatial distribution of GI is shaped by socio-economic structure, land values, and governance decisions, ultimately producing varying spatial justice outcomes in terms of vulnerability, resilience, and livability. Feedback loops mean these outcomes, in turn, influence future metabolic flows and governance priorities.

This framework positions spatial justice not as a secondary social issue but as an integral dimension of urban metabolic health. It directly guides the comparative NDVI analysis conducted in this study: rather than treating vegetation as merely an aesthetic or environmental feature, we interpret GI as part of the broader urban metabolic system and use the Disparity Ratio as an indicator of how unevenly metabolic benefits are distributed across income groups.

### Research design and case selection

This study employs a comparative case study design to examine relationships between green infrastructure distribution and urban metabolic characteristics across distinct urban contexts. Three cities were selected to represent different positions along several relevant dimensions: metabolic stress, governance capacity, and climatic context.

#### Amman, Jordan

Positioned as the primary arid city case, Amman represents a rapidly growing city in a water-scarce region with evolving governance approaches. With over 4 million metropolitan residents, Amman has experienced dramatic growth through successive waves of migration while facing chronic water shortages, extreme summer heat, and documented socio-spatial divides between its eastern and western districts^32,33^. Despite these significant metabolic challenges, Amman has not been subject to comprehensive urban metabolism assessment, representing both a knowledge gap and a research opportunity.

#### Cairo, Egypt

Selected as a comparative case of high-density arid city metabolism, Cairo has been included in several comparative UM studies^2^ and has documented socio-spatial fragmentation and vegetation disparities^34^. Its acute metabolic pressures, including water dependency on the Nile, extensive informal settlements, and fragmented infrastructure^30^, make it a valuable case of metabolic stress in a Global South megacity context. It is included not as a model of failure, but as a context where metabolic pressures and spatial inequities are particularly visible.

#### Amsterdam, Netherlands

Included as a reference case with different governance traditions and metabolic patterns. Amsterdam has implemented integrated planning approaches^35^, circular economy initiatives, and maintains relatively high overall vegetation cover.

It is not positioned as a normative ideal but as a context where metabolic flows and GI are more evenly distributed across socio-economic groups, enabling examination of how reduced spatial disparity relates to metabolic coherence.

It is important to acknowledge upfront that what counts as “high income” or “low income” differs substantially across these three countries given their very different standards of living and economic contexts. Jordan, Egypt, and the Netherlands operate on entirely different income scales. Throughout this analysis, income stratification is therefore relative and intra-city: zones are classified as high, medium, or low income relative to the distribution within each respective city, not in absolute or cross-national terms.

### Data sources and spatial stratification

**Fig. 2:**
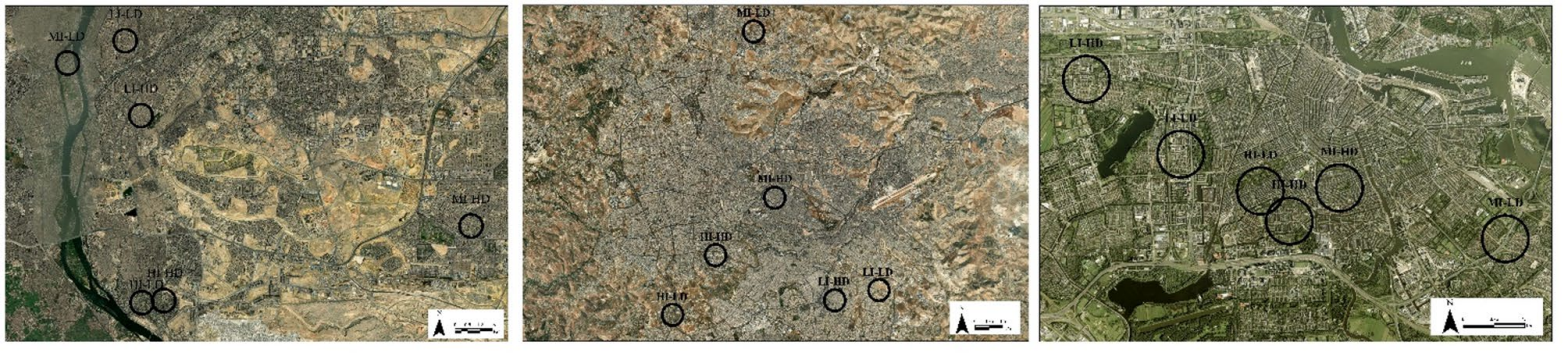
Overview map showing the six sampled socio-spatial zones in each city (Amman, Cairo, Amsterdam), classified by income level (high/medium/low) and density (high/low). Zone boundaries are based on census data and verified through satellite imagery, each zone is marked with income estimates and the second part being the density ( LI, HD for example is medium income and high density).

The analysis employed a stratified sampling approach to ensure systematic comparison across socio-economic gradients within each city. The methodological process involved the following stages:

- Socio-economic Stratification: Each city was divided into socio-spatial categories based on district-level data on land value per square Km or income and population density. Sources included Greater Amman Municipality statistics, the Central Agency for Public Mobilization and Statistics (Cairo), and Statistics Netherlands (CBS).
- Zone Selection: Six primary socio-spatial categories were identified per city — High/Medium/Low Income × High/Low Density — based on solid-void and floor-area ratios of distributed built areas.
- Vegetation Assessment: NDVI values for each zone were derived from 10-metre resolution Sentinel-2 satellite imagery. NDVI provides a standardized measure of vegetation presence and density (values range from − 1 to + 1, with higher values indicating denser vegetation). It should be noted that NDVI captures vegetation presence but not accessibility, quality, or usability of green spaces — this scope limitation is discussed further in Sect.  3.4.
- Income Stratification Gradient Analysis: Zones were grouped by income category, and average NDVI values were calculated per category.
- Here we calculate the result of each assessment by ending up with the Disparity Ratio (High Income NDVI / Low Income NDVI) provides a simple comparative metric where values closer to 1.0 indicate more equitable distribution and a better spatial justice/equity score in each city.
- Metabolic Contextualization: Distribution patterns were examined in relation to each city’s documented metabolic characteristics, drawing from existing UM literature (Fig. 2).

### Analytical limitations

Several methodological limitations must be acknowledged at the outset. First, reliance on NDVI captures vegetation presence and density but provides no information on the quality, type, usability, or physical accessibility of green spaces — dimensions critical to environmental justice analysis. Second, socio-economic stratification is constrained by the varying granularity and availability of census data across the three cities, potentially affecting the extreme precision of inter-city comparisons, this is why cross checking with land value was a good way to ensure having precise results of the distribution especially in Cairo and Amman. Third, the analysis represents a static temporal snapshot, unable to account for seasonal variation or historical trends in green cover.

## Results

### Amman: gradients in an arid city context

Amman’s vegetation analysis reveals clear distributional patterns across its socio-economic landscape. High-income neighborhoods, predominantly in the city’s western districts, recorded the highest mean NDVI values (0.1655 average), while low-income zones — often in eastern Amman or peripheral settlements — demonstrated the lowest vegetation density (0.1061 average). Mid-income areas showed intermediate values (0.1436). These patterns result in a Disparity Ratio of 1.56, indicating that high-income areas are approximately 56% greener than low-income areas.

**Fig. 3:**
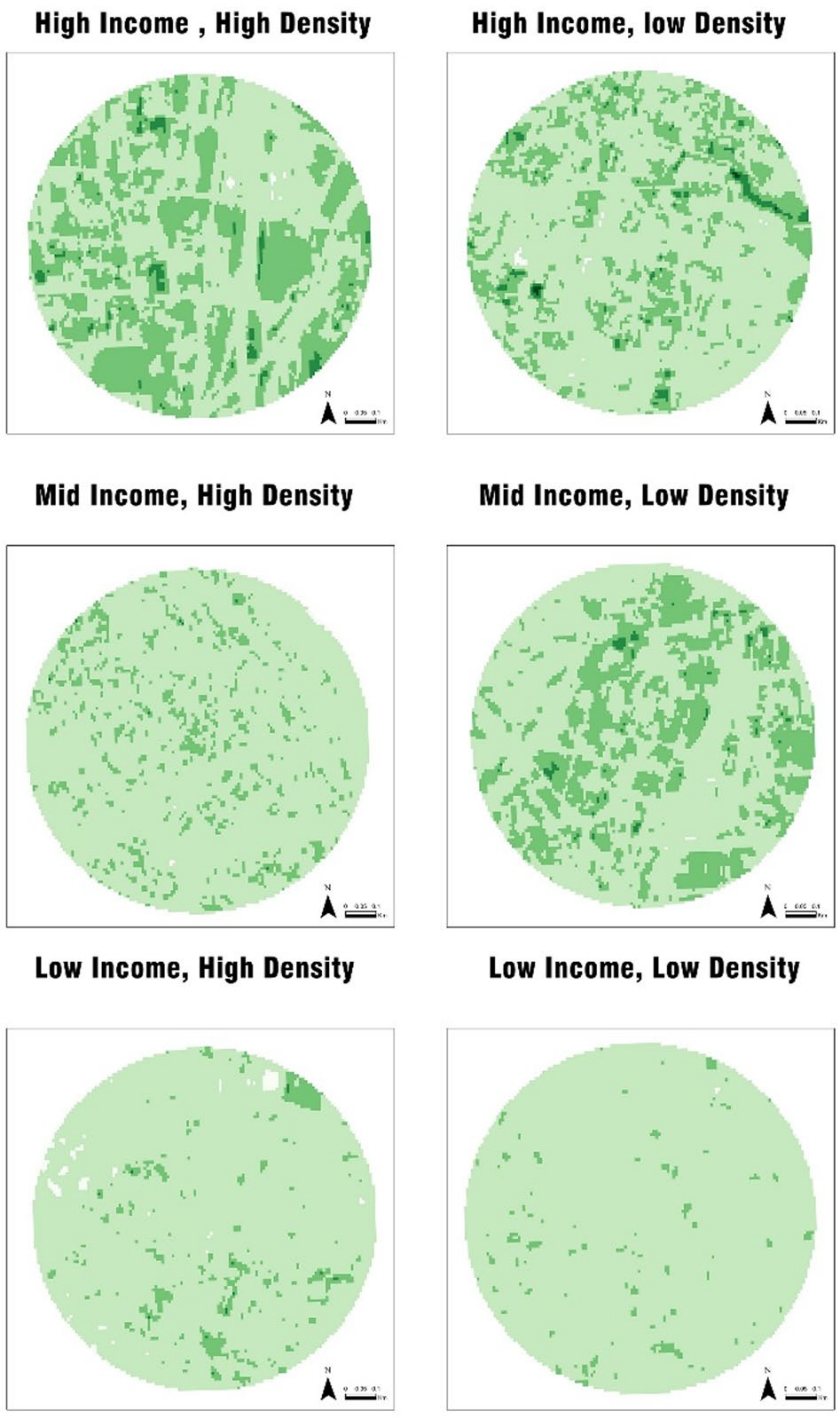
NDVI maps for all six sampled zones in Amman.

These gradients reflect Amman’s historical development trajectory: wealthier districts established on higher-elevation land zones have benefited from greater planning oversight and microclimatic advantages, while lower-income located in eastern neighborhoods face constrained vegetation alongside elevated ambient temperatures and reduced green space access. Notably, absolute vegetation levels are low across all zones (mean NDVI below 0.18), reflecting the arid climate context and water constraints. This limited absolute cover amplifies the significance of distributional disparities (Table 1; Fig. 3).

**Table 1:** NDVI metrics for amman by socio-spatial category.

Socio-economic Zone	Mean NDVI	STD	NDVI Range	NDVI Max	NDVI Min	Median	PCT90
High income, low density	0.1798	0.0905	0.6075	0.5934	0.0141	0.1790	0.2937
High income, high density	0.1511	0.0935	0.7702	0.7299	0.0403	0.1346	0.2743
Mid income, low density	0.1695	0.0815	0.5435	0.5362	0.0073	0.1648	0.2773
Mid income, high density	0.1177	0.0626	0.4770	0.4384	0.0386	0.1064	0.2773
Low income, low density	0.1181	0.0406	0.4020	0.3945	0.0075	0.1159	0.1705
Low income, high density	0.0941	0.0600	0.5344	0.4838	0.0506	0.0786	0.1727

### Cairo: pronounced disparities in a megacity context

Cairo’s vegetation distribution reveals the most pronounced socio-economic gradients of the three cities. High-income neighborhoods, including Zamalek and Heliopolis, show substantially higher vegetation density (mean NDVI 0.2671) compared to low-income areas (mean NDVI 0.0995), yielding a Disparity Ratio of 2.68. Mid-income zones occupy an intermediate position (mean NDVI 0.1500). These results indicate that high-income areas in Cairo are nearly 2.7 times greener than low-income areas — a gap that carries significant implications for differential exposure to urban heat and other environmental stresses.

**Fig. 4:**
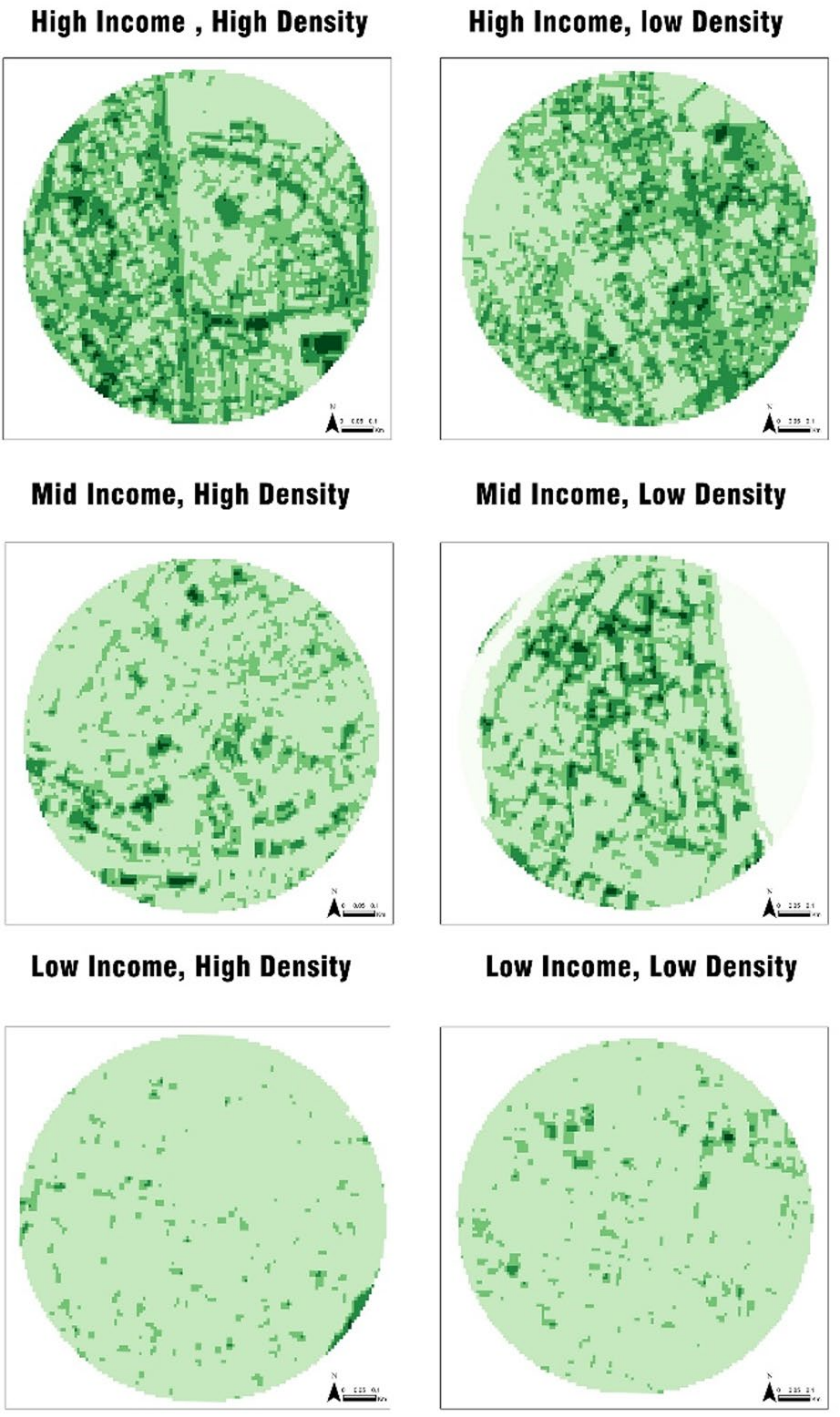
NDVI maps for all six sampled zones in Cairo.

Vegetation in Cairo operates as a “differentiated resource,” with high-income areas featuring private gardens, street trees, and maintained green spaces, while low-income districts face limited vegetation alongside other environmental challenges. An interesting pattern emerges in mid-income, high-density zones, which show the highest internal variability (standard deviation of 0.2158), reflecting heterogeneous development with mixed land uses in transitional urban areas (Table 2; Fig. 4).

**Table 2:** NDVI metrics for cairo by socio-spatial category.

Socio-economic Zone	Mean NDVI	STD	NDVI Range	NDVI Max	NDVI Min	Median	PCT90
High income, low density	0.2622	0.1441	0.7172	0.7459	0.0287	0.2444	0.4698
High income, high density	0.2720	0.1547	0.7059	0.7350	0.0290	0.2491	0.5004
Mid income, low density	0.1325	0.2158	1.0339	0.7201	−0.3138	0.1148	0.4369
Mid income, high density	0.1675	0.1154	0.6765	0.6874	0.0109	0.1309	0.3274
Low income, high density	0.0941	0.0606	0.6732	0.6638	−0.0094	0.0766	0.1498
Low income, low density	0.1048	0.0700	0.6503	0.6707	0.0204	0.0799	0.1874

### Amsterdam: attenuated gradients in a temperate context

Amsterdam presents a contrasting distribution pattern characterized by higher overall vegetation levels and more attenuated socio-economic gradients. High-income areas show mean NDVI values of 0.4523, while low-income areas remain relatively high at 0.4120, yielding a Disparity Ratio of 1.10. This suggests substantially more equitable GI distribution than in either arid city.

**Fig. 5:**
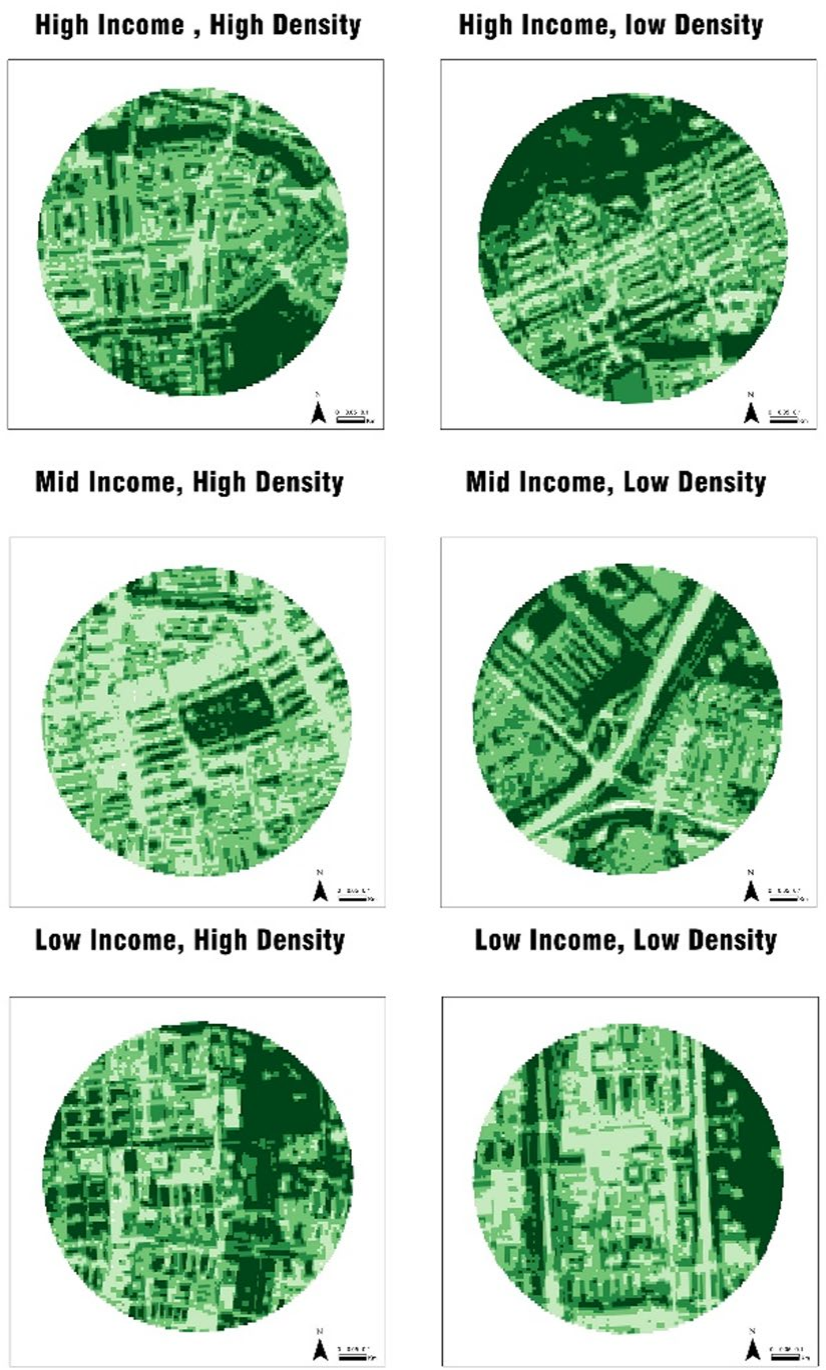
NDVI maps for all six sampled zones in Amsterdam. Greener tones indicate higher NDVI values. Note the relatively high vegetation coverage across all income categories compared to Amman and Cairo.

Notably, some low-income, high-density neighborhoods in Amsterdam demonstrate vegetation levels comparable to or exceeding those in wealthier districts. This likely reflects deliberate planning policies integrating parks and green corridors across the city, including in historically working-class areas. An exception appears in mid-income, high-density zones (mean NDVI 0.2994), possibly reflecting the challenges of greening dense historic central districts. Amsterdam’s overall higher vegetation levels (most zones above 0.30) reflect its temperate climate and fewer water constraints, enabling extensive greening regardless of neighborhood characteristics (Table 3; Fig. 5).

**Table 3:** NDVI metrics for Amsterdam by socio-spatial category.

Socio-economic Zone	Mean NDVI	STD	NDVI Range	NDVI Max	NDVI Min	Median	PCT90
High income, low density	0.4727	0.2176	0.9830	0.8779	−0.1051	0.4691	0.7779
High income, high density	0.4319	0.1924	0.8854	0.8394	−0.0461	0.4135	0.7072
Mid income, low density	0.4301	0.1908	0.9165	0.7899	−0.1265	0.4161	0.6958
Mid income, high density	0.2994	0.1790	0.8595	0.8024	−0.0570	0.2484	0.5914
Low income, high density	0.4446	0.2160	0.9047	0.8749	−0.0299	0.4320	0.7478
Low income, low density	0.3794	0.2104	0.8299	0.8326	0.0026	0.3401	0.6968

### Comparative analysis: disparity ratios in context

Synthesizing the city-level findings through the Disparity Ratio reveals distinct distributional patterns across the three cities. Cairo shows the most pronounced disparity, with high-income areas approximately 2.68 times greener than low-income areas. Amman occupies an intermediate position (1.56), while Amsterdam demonstrates the most equitable distribution pattern (1.10).

Crucially, these ratios exist within different absolute vegetation contexts. Amsterdam’s “low” income mean NDVI (0.4120) substantially exceeds Amman’s “high” income mean NDVI (0.1655), underscoring that both absolute availability and relative distribution shape urban environmental experiences. The Disparity Ratio is best interpreted as an intra-city equity indicator rather than a basis for direct cross-city comparison of environmental quality (Table 4).

**Table 4:** Comparative vegetation distribution metrics.

City	High Income NDVI	Mid Income NDVI	Low Income NDVI	Disparity Ratio (High: Low)
Amman	0.1655	0.1436	0.1061	1.56
Cairo	0.2671	0.1500	0.0995	2.68
Amsterdam	0.4523	0.3648	0.4120	1.10

## Discussion

### Interpreting distribution patterns across cities

Cairo’s pronounced disparities (Disparity Ratio 2.68) reflect what urban political ecology scholars characterize as “metabolic fracture” — a condition where environmental benefits become concentrated in privileged areas while burdens accumulate in marginalized ones^6,17^. This pattern intersects Cairo’s documented metabolic challenges, including water stress, air pollution, and reliance on informal waste systems^36^. Rapid informal expansion, speculative real-estate development, and state-led megaprojects have produced a fragmented urban fabric in which low-income districts experience chronic deficits in green space and environmental quality, while wealthier enclaves benefit from privatized greenery and infrastructure.

Amman’s intermediate Disparity Ratio (1.56) suggests a city in metabolic transition, where spatial inequality persists but is less extreme than in Cairo. The city’s east–west development history — with wealthier districts established on western hillsides through successive waves of migration and planning intervention — has created enduring socio-spatial patterns that continue to shape environmental quality distribution^32,33^. Amman’s intermediate position reflects a context where emerging metabolic improvements in some areas coexist with persistent spatial injustice in others.

Amsterdam’s attenuated disparities (1.10) reflect different historical trajectories and planning traditions. The city’s commitment to integrated green space planning, as exemplified in its Green Structure Plan, has promoted more equitable distribution of vegetation benefits. However, equitable distribution represents only one dimension of spatial justice: concerns about green gentrification and displacement pressures remain relevant even in cities with relatively equitable vegetation distribution^25^.

### Spatial justice as a diagnostic tool for urban metabolic performance

This research reframes spatial justice from a primarily normative concern into a measurable diagnostic lens for evaluating urban metabolic performance. The Disparity Ratio functions not as a definitive measure of justice or sustainability, but as an indicative metric that highlights the degree to which metabolic benefits (cooling, ecological buffering, environmental comfort) are unevenly distributed across socio-economic groups. As a diagnostic tool, the approach enables cities to conduct a rapid assessment of potential metabolic imbalance without relying solely on comprehensive city-wide material flow analysis. Elevated disparity values should therefore be understood as early-warning signals of metabolic stress, suggesting heightened vulnerability and reduced resilience on the neighborhood scale.

The relationship between vegetation distribution and broader urban metabolic characteristics is multifaceted and context dependent. In arid cities, vegetation distribution intersects critically with water metabolism: areas with greater greenery typically require more irrigation, creating trade-offs between local environmental quality and regional water security^27^. If energy-saving vegetation benefits concentrate in already-privileged areas, this reinforces existing inequalities in energy burden and thermal comfort — a particularly acute concern in arid climates where cooling is essential.

The findings also suggest connections between distribution patterns and governance approaches to urban metabolism. Amsterdam’s integrated planning and circular economy initiatives align with more equitable vegetation distribution, though establishing causal direction would require longitudinal analysis. Cairo’s more fragmented governance, with stark divisions between formal and informal development, corresponds with pronounced vegetation disparities^34,36^. Amman’s evolving governance, balancing rapid growth pressures with sustainability ambitions, is reflected in its intermediate pattern^33^.

From a policy perspective, these findings suggest that investments in GI within underserved neighborhoods should be interpreted as strategic metabolic interventions, not merely as redistributive or welfare-oriented measures. In Amman specifically, targeted greening of eastern low-income, high-density districts — combined with water-efficient species selection and governance reforms that prioritize public green space — could simultaneously address equity deficits and reduce metabolic vulnerability. In Cairo, the scale of the disparity points to the need for systemic planning reform, including mandatory green space standards in informal settlement upgrading programs. In Amsterdam, the priority may be to ensure that continued densification and green gentrification pressures do not erode the relatively equitable GI distribution achieved to date.

### Methodological contributions and limitations

This research demonstrates the value of integrating spatial distribution analysis into urban metabolism studies. The methodology — combining NDVI assessment with socio-economic stratification and Disparity Ratio calculation — provides a replicable approach for examining distributional dimensions across diverse urban contexts. It complements traditional material flow analysis with spatial equity diagnostics and is particularly suited to data-scarce environments where comprehensive MFA is not feasible.

Several limitations warrant acknowledgement. Vegetation distribution represents only one dimension of green infrastructure and environmental justice; accessibility, quality, tree species diversity, and maintenance regimes are not captured through NDVI alone. The Disparity Ratio simplifies complex distributional patterns into a single metric, potentially obscuring important spatial nuances. The comparative approach faces inherent challenges due to differences in data availability, historical trajectories, and cultural contexts. Future research should address these limitations by incorporating accessibility measures, longitudinal data, and qualitative governance analysis.

## Conclusion

This research has examined the relationship between spatial justice and urban metabolism through comparative analysis of green infrastructure distribution in Amsterdam, Cairo, and Amman. Our findings reveal distinct vegetation distribution patterns, with Disparity Ratios of 1.10 (Amsterdam), 1.56 (Amman), and 2.68 (Cairo) quantifying varying degrees of socio-spatial inequality.

These patterns relate to broader urban metabolic characteristics and governance contexts. Cairo’s pronounced vegetation disparities intersect with its documented metabolic challenges and fragmented urban development^34,36^. Amman’s intermediate disparities reflect arid climate constraints and transitional governance^32,33^. Amsterdam’s more equitable distribution aligns with integrated planning traditions and circular economy initiatives^2,9^.

The study’s principal contributions are threefold. Methodologically, it integrates spatial distribution analysis into UM studies through a replicable, accessible approach. Theoretically, it positions spatial justice as an integral dimension of urban metabolic health rather than a secondary social concern, advancing integrated frameworks^4–6^. Empirically, it provides insights from Amman as an underrepresented case of arid city metabolism, offering comparative perspective from Cairo and Amsterdam.

Several directions for future research emerge. Studies could examine how vegetation distribution patterns evolve over time in relation to policy interventions. More comprehensive assessments could incorporate additional dimensions of GI beyond vegetation density, including accessibility and multi-functionality^23,25^. Research could also explore specific mechanisms connecting distribution patterns to metabolic outcomes such as energy use, water consumption, and public health indicators^19,21,27^. Finally, future work should seek to directly measure metabolic flows at the neighborhood scale alongside GI distribution metrics, enabling more definitive causal analysis.

For cities confronting rapid urbanization and climate pressures (particularly in arid regions) this research underscores that distributional dimensions are not secondary to metabolic efficiency but integral to it. Equity in access to green infrastructure is both a social justice imperative^14,16^ and a determinant of urban resilience and livability^20,23^. As cities worldwide face intersecting challenges of climate change, resource constraints, and inequality, approaches that bridge metabolic and justice considerations will become increasingly essential. Spatial justice requires greater attention within urban metabolism scholarship and practice.

## Supplementary Information

Below is the link to the electronic supplementary material.

Supplementary Material 1

## Data Availability

All data generated or analyzed during this study are included in this published article and its Supplementary Information files.
